# Transduction of dendritic cells with recombinant adenovirus encoding HCA661 activates autologous cytotoxic T lymphocytes to target hepatoma cells

**DOI:** 10.1038/sj.bjc.6601706

**Published:** 2004-03-23

**Authors:** R C-F Chan, X-W Pang, Y-D Wang, W-F Chen, Y Xie

**Affiliations:** 1 Department of Biology, The Hong Kong University of Science and Technology, Clear Water Bay, Kowloon, Hong Kong SAR, China; 2 Department of Immunology, Peking University Health Science Centre, Beijing, 100083, China

**Keywords:** hepatocellular carcinoma, hepatoma, liver cancer, HCA661, dendritic cell, recombinant adenovirus, cytotoxic T lymphocytes, tumour vaccine

## Abstract

Transduction of recombinant adenovirus into dendritic cells (DCs) is a promising new tool for cancer vaccine development. Here, we report that an adenovirus vector carrying hepatocellular carcinoma (HCC) antigen HCA661 and infected into DCs generates T-cell immunity against hepatoma cells. HCA661 is a novel cancer/testis (CT) antigen screened by SEREX from sera of an HCC patient. We constructed a recombinant adenovirus expressing the full-length cDNA of HCA661 gene and then transduced immature DCs, which had been generated with GM-CSF and IL-4 from peripheral blood mononuclear cell of HLA-A2^+^ healthy donors. The resulting adenovirus-transduced DCs differentiated in the presence of monocyte-conditioned medium and poly [I] : poly [C], expressing the surface markers of mature DCs, including CD83, CD80, CD86 and HLA-DR. After maturation, the transduced DCs transcribed HCA661 mRNA and were able to prime the naïve T cells to become cytotoxic T lymphocytes (CTLs). Intracellular flow cytometry and enzyme-linked immunospot assay showed that these CTLs were able to target a hepatoma cell line, HepG2, which is HLA-A2 and HCA661 positive. In summary, we found that this recombinant adenovirus can help to induce DC maturation and these mature DCs can activate T cells to target hepatoma cells. Therefore, this recombinant adenovirus may have potential for use in liver cancer immunotherapy.

Hepatocellular carcinoma (HCC) is the third most common cause of cancer death in China (Stern *et al*, 2001). Hepatocellular carcinoma is one of the most deadly cancers, rarely responding to conventional treatment such as radiation therapy or chemotherapy, and usually causing death within a few weeks or months of detection after diagnosis (Tang, 1989).

Transduction of recombinant adenovirus into dendritic cells (DCs) is a promising new tool for cancer vaccine development. Most cancer cells are poor immunogens due to the lack of costimulatory element B7 or the downregulation of MHC class I expression, both of which are essential for activation of T cells (Gong *et al*, 2000). Therefore, cancer cells do not activate the T-cell response. On the other hand, DCs are potent antigen-presenting cells that prime naive cytotoxic T lymphocytes (CTLs) in an HLA-restricted fashion (Inaba *et al*, 1986; Steinman, 1991) and have been shown to induce potent antitumour immunity *in vitro* and *in vivo* (Timmerman and Levy, 1999). Immature DCs such as Langerhans cells (Romani *et al*, 1989; Sallusto and Lanzavecchia, 1994) are highly specialised in antigen capture, whereas mature DCs are migratory cells that play a major role in antigen presentation (Steinman *et al*, 1995; Morse *et al*, 2001). Mature DCs downregulate endocytotic activity and upregulate MHC class II and costimulatory molecules, thus enhancing antigen-presenting capacity for the promotion of T-cell activation. One effective method of enabling DCs to activate CTL response against cancer cells is to transfect cancer antigens into DCs via recombinant adenovirus (Linette *et al*, 2000). Recombinant adenoviral vectors have become common in the transduction of tumour antigens into terminally differentiated and nondividing DCs (Dietz and Vuk-Pavlovic', 1998) because of their high efficacy in inducing both humoral and cell-mediated immune responses (Ertl and Xiang, 1996).

In this study, we used the adenoviral construct based on a E1- and E3-deleted replication-defective virus of the human strain 5 (Wilson *et al*, 1994) fused with HCC antigen HCA661. HCA661 is a novel cancer/testis (CT) antigen screened by SEREX from sera of HCC patients (Wang *et al*, 2002). Cancer/testis antigens are predominantly expressed in cancer tissues, but not in normal counterparts except in germ cells in testis or ovary (Wang *et al*, 2002), which has made them very useful in vaccine-based immunotherapy (Rosenberg, 1999).

Here, we report that (1) recombinant adenovirus (Ad661) was able to transduce human immature DCs, (2) DCs cultured with monocyte-condition medium (MCM) and poly [I] : poly [C] developed phenotypically into mature phenotypes, (3) the mature DCs expressed full-length HCA661 mRNA and (4) these mature DCs stimulated HCA661-specific CTL response. We demonstrate that this recombinant adenovirus Ad661 may be a promising compound for use in liver cancer immunotherapy.

## MATERIAL AND METHODS

### Culture of HCC cell lines and isolation of peripheral blood mononuclear cells (PBMCs)

Human HCC HepG2, SMMC-7721, BEL-7402 and QGY-7703 cells were cultured in RPMI 1640 medium (GIBCO-BRL, USA) supplemented with 10% heat-inactivated FBS (GIBCO-BRL, USA), 2 mM L-glutamine (GIBCO-BRL, USA), 100 U ml^−1^ penicillin and 100 *μ*g ml^−1^ streptomycin (GIBCO-BRL, USA).

Peripheral blood mononuclear cells were isolated from freshly collected buffy coats of healthy donors (Hong Kong Red Cross Association) by Ficoll–Hypaque (1.077 g; Amersham Biosciences, USA) density gradient centrifugation. The HLA typing of donor PBMCs was carried out by staining with a mouse anti-HLA-A2 antibody (BB7.2, Amersham Biosciences, USA) using standard procedures. The PBMCs were washed with PBS and resuspended in RPMI 1640 supplement with 10% FBS and plated in six-well tissue culture plates (Falcon, USA) at a density of 2 × 10^6^ cells well^−1^ and incubated at 37°C for 2 h prior to incubation.

### Preparation of mature dendritic cells and human T cells

After 2 h incubation, the nonadherent PBMCs were removed by washing with PBS, and autologous T cells were isolated from these nonadherent PBMCs by nylon-wool separation according to the manufacturer's protocol (Polysciences, USA). The adherent cells were cultured for 7 days in RPMI 1640 medium supplemented with 10% FBS, 1000 U ml^−1^ human granulocyte–macrophage colony-stimulating factor (hGM-CSF; Sandoz Pharma Ltd, China) and 500 U ml^−1^ hIL-4 (Peprotech, UK). Culture medium and cytokines were refreshed every other day. On day 7, the nonadherent and loosely adherent cells were harvested by repeated washes to generate the immature autologous DC population.

### Generation of recombinant adenovirus encoding HCA661

To subclone the human HCA661 cDNA, the plasmid pQE3.1/HCA661 (Wang *et al*, 2002) was used as a template for polymerase chain reaction (PCR) using specific oligonucleotides encompassing the entire open reading frames. The following PCR primers were used: human Ad661 sense 5′-CGGAATTCATGGCAAAATATGTC AGT-3′, human Ad661 antisense 5′-GCTCTAGATTAGTCATCCTCGTCATTC TC-3′. Polymerase chain reaction was performed with the following profile: 60 s at 95°C, 60 s at 60°C and 90 s at 72°C for 30 cycles, followed by a last 10 min extension at 72°C. The amplified products were cloned into the *Eco*RI/*Xba*I sites of pCR-259 adenovial transfer vector, derived from adenovirus type 5 with deleted E1 and E3 regions (Qbiogene, USA) and then generated the recombinant adenovirus encoding HCA661 (Ad661), according to the protocol of transposed-Ad adenoviral system stated by the manufacturer (Qbiogene, USA). The recombinant adenovirus was amplified using QBI-HEK 293 cells (Qbiogene, USA) and the concentration of the viral titer obtained was 2 × 10^9^TCID_50_ ml^−1^.

### Transduction of DCs with recombinant adenovirus

The immature DCs (1 × 10^5^) obtained on day 7 were resuspended in 50 *μ*l cytokine-containing medium and placed as a small drop into one well of 24-well plates. Recombinant adenovirus was added at 1000 multiplicity of infection (MOI). The mixture was incubated at 37°C for 2 h. Then, the culture medium obtained from day 7 culture containing GM-CSF and IL-4, 25% (v v^−1^) MCM and 10 *μ*g ml^−1^ poly [I] : poly [C] (Sigma, USA) was added to make a total volume of 500 *μ*l. The adenovirus QBI-Infect+ (Qbiogene, USA) containing the lacZ gene for *β*-galactosidase was used as a negative control. On day 9, the nonadherent and loosely adherent cells were harvested by repeated washing and the phenotype of the mature autologous DCs was identified. Transduction by QBI-Infect+ was measured by the hydrolysis of Bluo-gal (Invitrogen, USA). The transfected DCs were washed and irradiated at 30 Gy *γ*-irradiation using *γ*-cell irradiator (Gamma-Elite1000). The irradiated cells were stored at −80°C for presensitisation of T cells. The expression of HCA661 gene from transfected DCs was measured by reverse transcription–polymerase chain reaction (RT–PCR).

### Generation of MCM

Plates (100 mm; Falcon, USA) were precoated with an immunoglobulin (Ig) by adding 5 ml of human *γ*-globulin (10 mg ml^−1^; Calbiochem, USA) for 1 h at 37°C. After washing with PBS, PBMCs (1 × 10^7^) in 10 ml of complete culture medium were added to the Ig-coated plates and incubated for 1 h at 37°C. Nonadherent cells were removed and the remaining adherent cells were cultured in fresh complete medium at 37°C for 24 h. Monocyte-conditioned media were centrifuged to remove the cell debris and then frozen at −20°C prior to use.

### Flow cytometry

Dendritic cells were collected by centrifugation at 1500 r.p.m. for 10 min and washed with PBS twice. The cells were incubated with murine mAbs targeting CD83 (Pharmingen, USA), HLA-DR (MHC class II; Pharmingen, USA), B7-1 (Pharmingen, USA), B7-2 (Pharmingen, USA) or ICAM-1 (Pharmingen, USA) on ice for 1 h. After washing the cells with PBS twice, the cells were incubated with FITC-labelled goat anti-mouse IgG antibody (1 : 50; Zymed, USA) and incubated for 30 min on ice. After washing with PBS twice, the cells were resuspended in 0.5 ml 2% paraformaldehyde (Sigma, USA). The fixed cells (10^5^ cells per event) were analysed by FACScan (Becton Dickinson, USA). Cells without the primary Ab staining antibody were used as a negative control.

### Endocytosis assay

Endocytic activity was assessed by measuring uptake of the fluid phase marker FITC-dextran (MW 4000; Sigma, USA). Immature or mature DCs (1 × 10^5^) were incubated at 37°C in a complete medium containing 1 mg ml^−1^ FITC-dextran. A control experiment was performed by incubating the cells at 4°C for 1 h. After incubation, the cells were washed twice with ice-cold PBS and then subjected to flow cytometry analysis (Bell *et al*, 2001).

### Reverse transcription–polymerase chain reaction

The HCA661 mRNA expression of the HepG2 cells and transfected DCs was assayed by RT–PCR. The mRNA from the cells was extracted according to the protocol of the mRNA extraction kit (Roche, Germany). The mRNA was primed with a dT_(18)_ oligonucleotide at 70°C for 5 min and put on ice for 2 min. The mRNA was reverse transcribed into cDNA using 200 U M-MLV reverse transcriptase (Promega, USA) at 42°C for 1 h. HCA661 gene-specific PCR primers were used to amplify the HCA661 fragments of 1kb in length. The following PCR primers were used: human HCA661 sense 5′-CGGAATTCATGCCTCAGAGACCA-3′ and human HCA661 antisense 5′-GCTCTAGATCAGTCATCCTC-3′. The PCR products were visualised in 1% agarose gel electrophoresis.

### *In vitro* sensitisation with recombinant adenovirus constructs

The transfected DCs (1 × 10^5^) were washed and cocultured with HLA-A2-positive T cells (1 × 10^6^ cell ml^−1^) from the same donor in 24-well plates (Falcon, USA). Cells were subcultured twice a week with complete medium containing IL-2 (20 U ml^−1^) and IL-7 (10 ng ml^−1^; Peprotech, UK). The irradiated transfected DCs were added to the plates every week. After incubation for 20 days, the activated T cells were used as effector cells for intracellular staining and IFN-*γ*-producing enzyme-linked immunospot ELISPOT assay.

### Intracellular staining for IFN-*γ*

The presensitised T cells obtained from one healthy donor were cocultured with irradiated HepG2 cells at an effector/target ratio of 10 : 1 for 6 h at 37°C in the presence of 2 *μ*M monensin A (ebioscience, USA). After incubation, the cells were harvested, washed with PBS, and stained with FITC-conjugated anti-CD8 (Pharmingen, USA) for 30 min at 4°C. After washing, the cells were fixed with 2% paraformaldehyde, and washed once with FACS buffer and once with permeabilisation buffer (0.1% saponin in PBS). Subsequently, the cells were stained with PE-conjugated anti-IFN-*γ* antibody (Pharmingen, USA) for 30 min at 4°C. Cells were washed once with permeabilisation buffer and once with FACS buffer, and then analysed by flow cytometry. Peripheral blood mononuclear cells obtained from normal volunteers incubated with 10 *μ*g ml^−1^ of phytohemagglutinin (PHA) were used as a positive control.

### Enzyme-linked immunospot assay

A mAb against human IFN-*γ* (NIB42; Pharmingen, USA) at 10 *μ*g ml^−1^ diluted in carbonated buffer (pH9.5) was coated onto flat-bottomed 96-well nitrocellulose plates (MultiScreen-HA, Millipore, USA), and incubated overnight at 4°C. After washing with PBS, plates were blocked with RPMI 1640 medium with 10% FBS for 2 h at 37°C. HepG2 cells irradiated at 30 Gy were applied as target cells in this experiment. Presensitised T-effector cells (4 × 10^5^) from two different HLA-A2^+^ healthy donors and irradiated target cells (5 × 10^4^) in complete medium were added to the plates and incubated for 24 h at 37°C. Plates were then washed thoroughly with PBS-Tween 20. A biotinylated anti-IFN-*γ* antibody (4S.B3; Pharmingen, USA) at a concentration of 1 *μ*g ml^−1^ was added and incubated at room temperature for 2 h. After washing with PBS-Tween six times, a streptavidin–HRP conjugate antibody (Zymed, USA) at a 1 : 2000 dilution was added and incubated at room temperature for 1 h. Following washing with PBS-T six times, spots were developed by adding an AEC substrate (Sigma, USA) and incubated for 15–20 min at room temperature. Plates were washed with distilled water and air dried. The spots were then counted under the dissected microscope.

### Statistical analysis

Statistical analysis of the spots obtained from ELISPOT assay was performed with a two-tailed Student *t*-test. The values of *P*<0.05 were regarded as significant.

## RESULTS

### Maturation of DCs transfected with recombinant adenovirus in MCM and poly [I] : poly [C]

**Figure 1:**
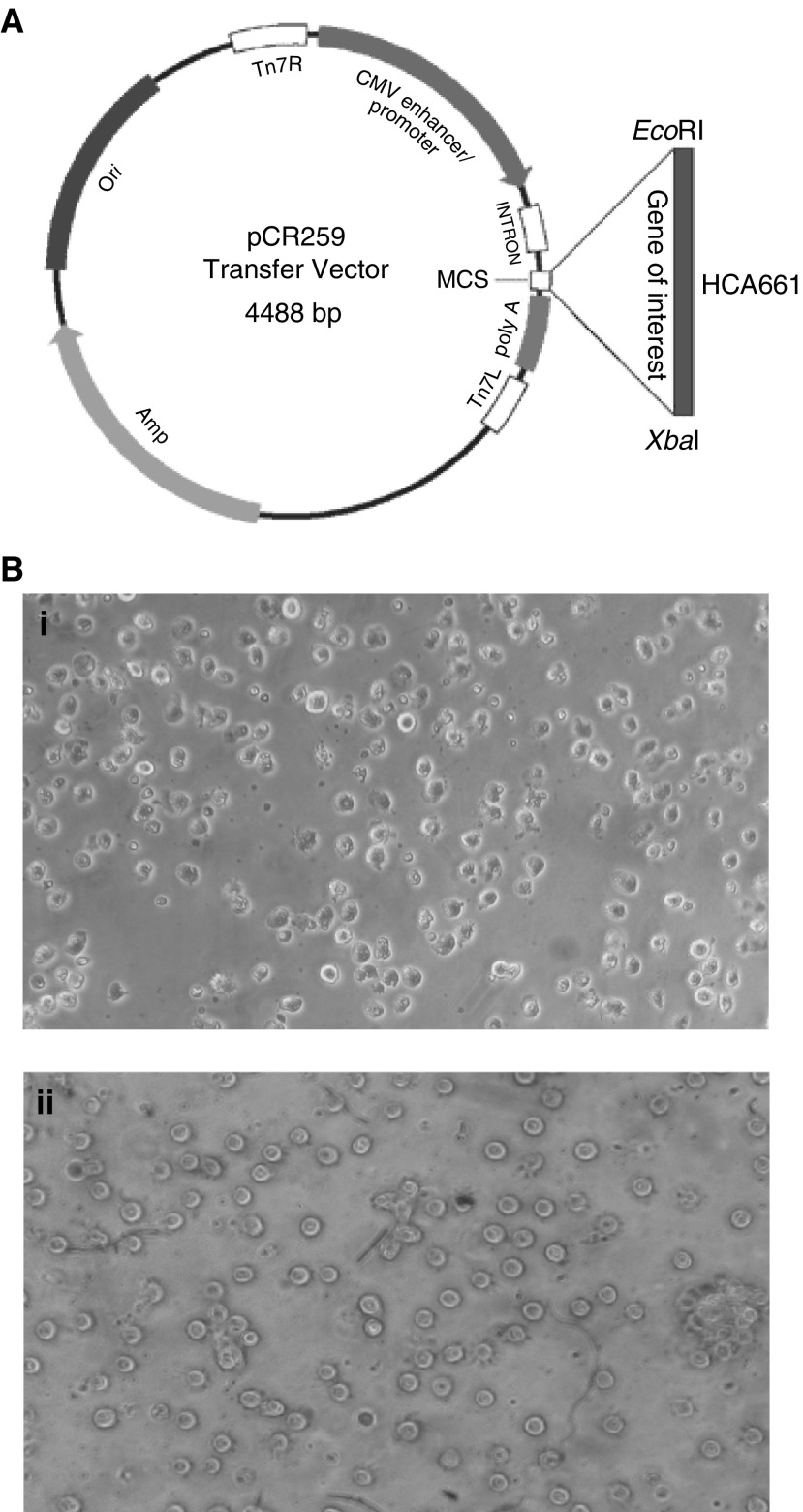
(**A**) Construction of recombinant adenoviruses (Ad661). HCA661 was cloned into *Eco*RI/*Xba*I sites of an adenoviral transfer vector pCR259 (Qbiogene). (**B**) (i)Transfection of day 7 immature human DCs that had been cultured in GM-CSF and IL-4 by QBI-Infect+ (AdenoLacZ) at an MOI of 1000. Transfected DCs were then cultured for 48 h in MCM and poly [I] : poly [C] for maturation. *β*-Galactosidase was measured with Bluo-gal substrate. (ii) DCs matured in MCM and poly [I] : poly [C] without adenovirus transfection.

**Figure 2:**
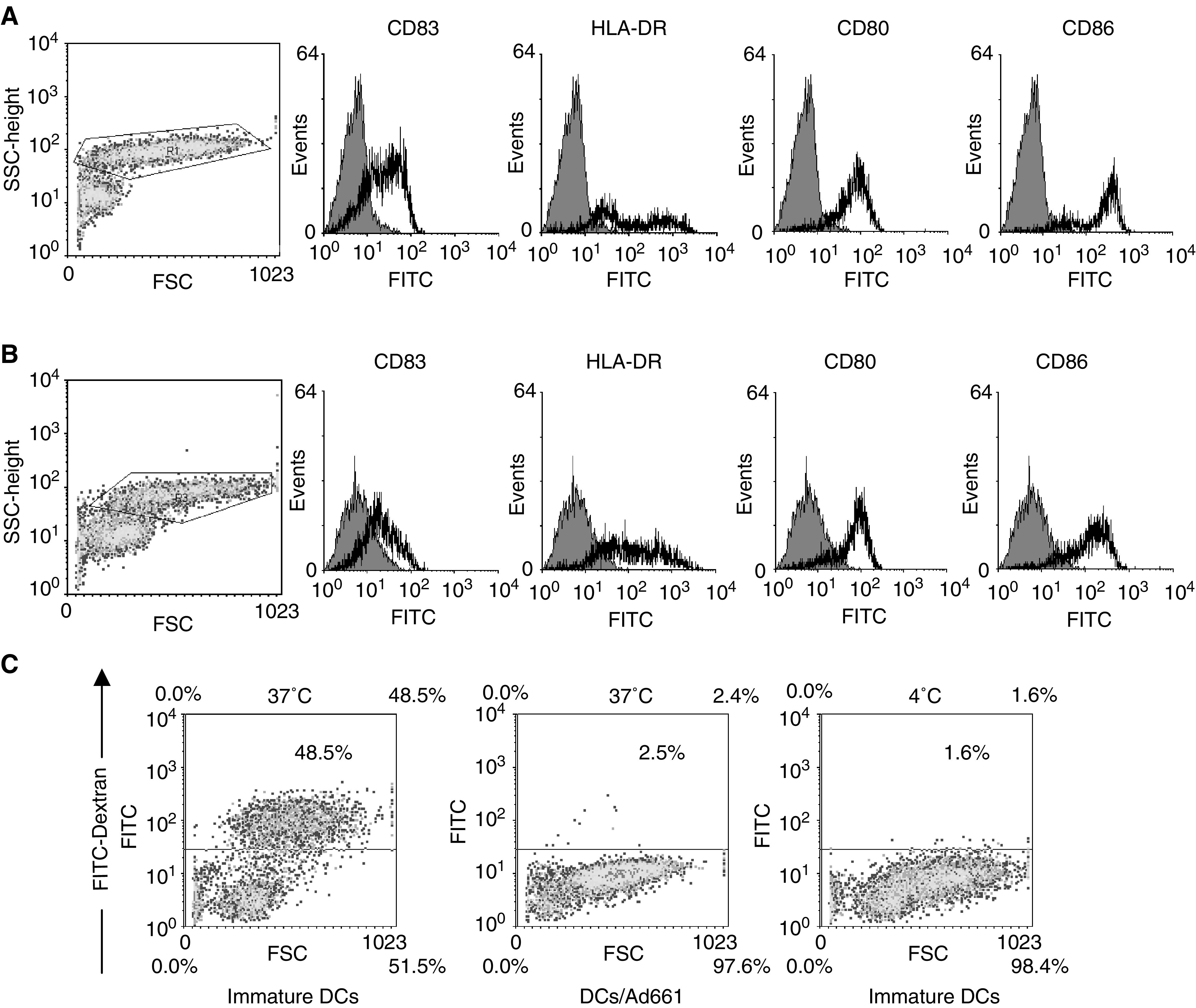
Monocytes incubated with GM-CSF and IL-4 developed immature DCs on day 7. After infection with (**A**) recombinant adenovirus (Ad661) or (**B**) without infection, and incubation with MCM and poly [I] : poly [C], the mature DCs were developed within 48 h. The expression of DC maturation markers was determined by FACS with the cells gated in the scatter profile. (**C**) The DCs (have also been transfected by Ad661) decreased their endocytosis ability after maturation. Endocytosis was determined by analysis of FITC-dextran internalisation at 37 and 4°C (negative control).

**Figure 3:**
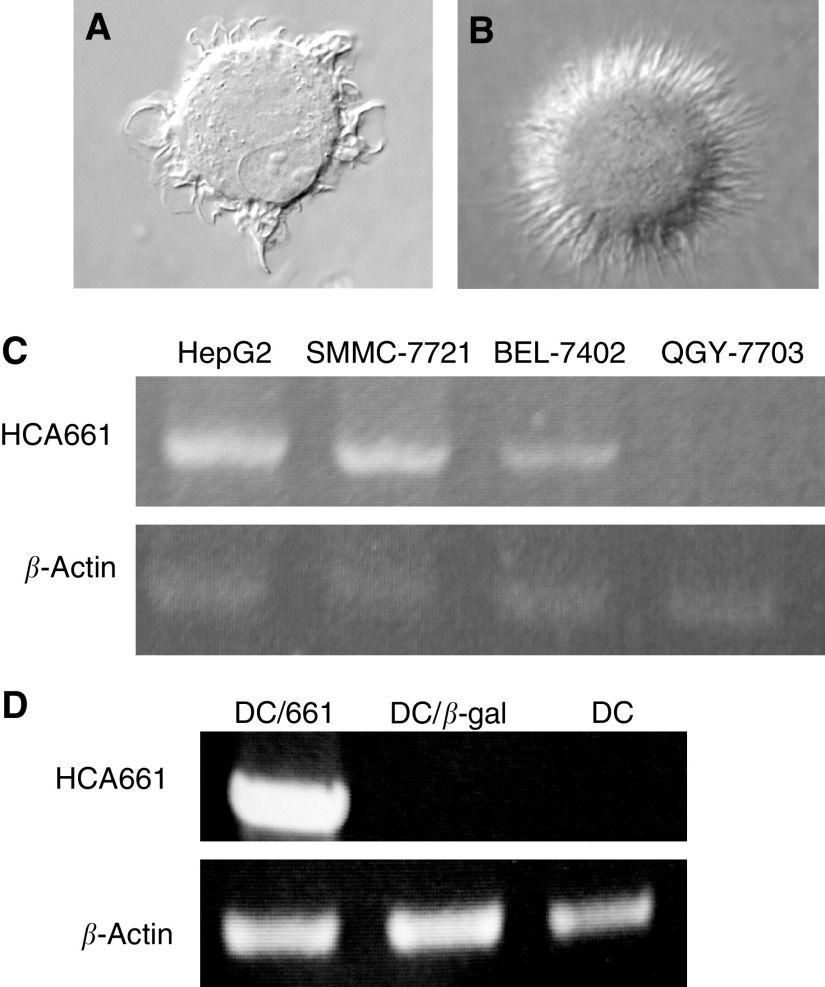
Cell morphology of (**A**) immature DCs and (**B**) mature DCs (have also been transfected by Ad661) was assessed by microscopy at 100 × magnification. (**C**) Expression of HCA661 mRNA in four HCC cell lines was analysed by RT–PCR. Gel electrophoresis of RT–PCR products shows HCA661 transcripts in HepG2, SMMC-7721 and BEL-7402 cells. Reverse transcription–PCR for *β*-actin was used to monitor the quality of the mRNA sample and act as an internal control. (**D**) Expression of the transgene HCA661 in the transduced DCs on day 9 was determined by RT–PCR. Only the DCs transduced by Ad661 show HCA661 mRNA transcripts; DCs alone and DCs (DC/*β*-gal) transduced by QBI-Infect+ did not express HCA661.

After 7 days culture of adherent monocytes in GM-CSF and IL-4, immature DCs were generated (Figure 3A) and transfected by recombinant adenovirus. The transfected DCs were then cultured 2 days in medium containing maturation stimulants, MCM and poly [I] : poly [C]. Most transfected DCs expressed high levels of CD83 (a maturation marker for DCs), HLA-DR, CD80 and CD86 (Figure 2A). The expression of these surface antigens was comparable to nontransfected controls (Figure 2B). Also the morphology of the mature DCs showed more and longer dendrites protruding out from the cell surface (Figure 3B). The endocytosis ability of transfected DCs was downregulated (Figure 2C). Our data showed that the maturation of DCs was not inhibited by adenoviral transduction. In the control DCs expressing the mature phenotype, over 90% of the cells expressed *β*-galactosidase when transfected with QBI-Infect+ at 1000 MOI as indicated by Bluo-gal staining (Figure 1B

, Figure 2

). Thus, the rate of transduction of DCs by recombinant adenovirus was high.

### HCA661 mRNA expression in Ad661-transfected DCs

To determine the transcription of HCA661 mRNA, RT–PCR was performed on RNA obtained from Ad661-transfected DCs. Dendritic cells were transfected with Ad661 or QBI-Infect+ at MOI 1000 and cultured for 48 h, at which time RNA was extracted. In DCs transfected with Ad661, HCA661 mRNA was detected (Figure 3D

). In contrast, HCA661 mRNA was not detected in DCs transfected with QBI-Infect+ (Figure 3D). A *β*-actin fragment served as an internal control. In addition, HCA661 mRNA was also endogenously expressed in HepG2 cells as shown in RT–PCR (Figure 3C).

### Activation of HCA661-specific CTLs by Ad661-transfected DCs

To generate a specific CTL response against HCA661 expressed on HepG2 cells, PBMCs from HLA-A2^+^ donors were stimulated with irradiated DCs transfected by Ad661or QBI-Infect+.

**Figure 4:**
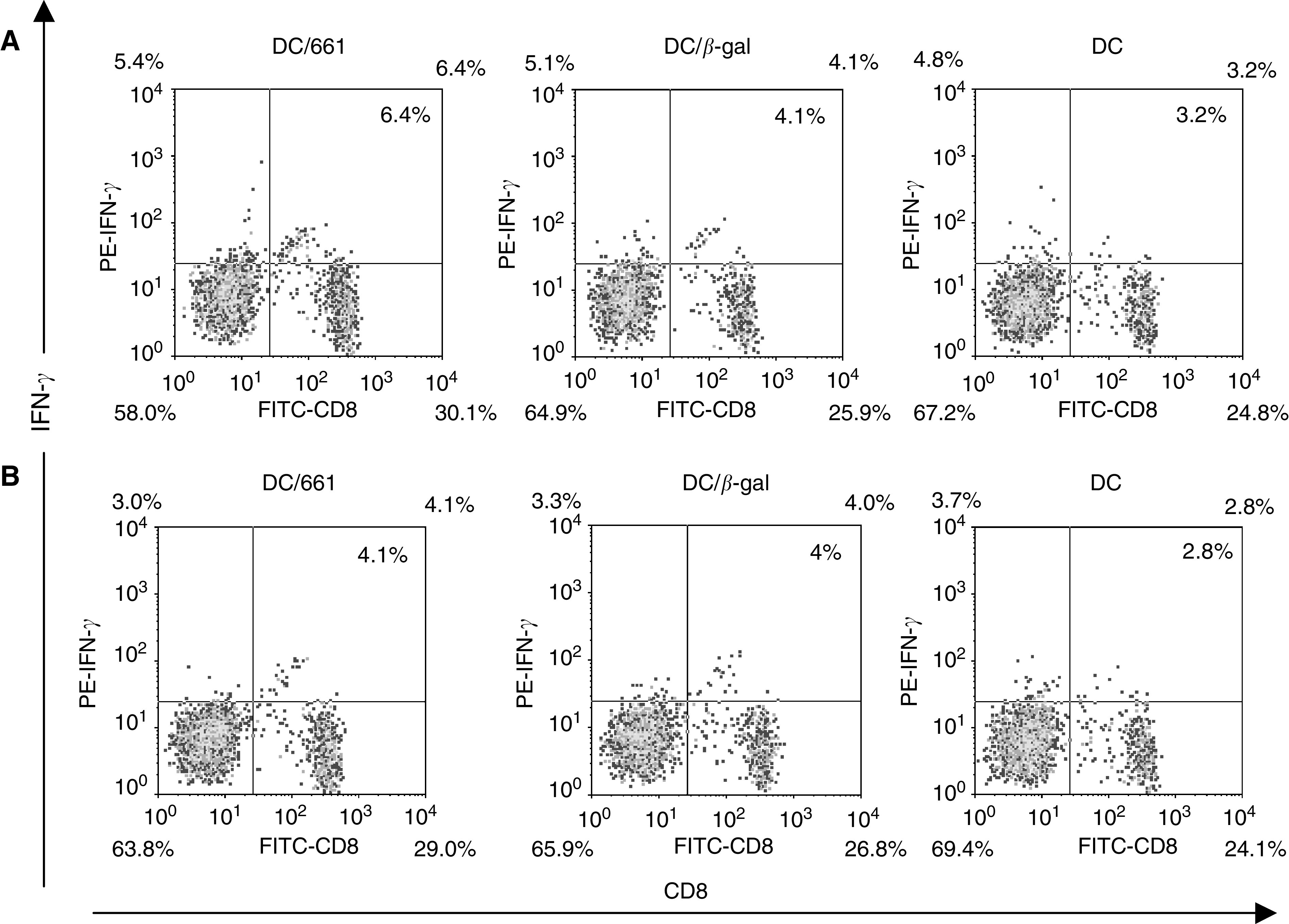
Intracellular flow cytometry assay for detection of IFN-*γ*-expressing CD8^+^ T lymphocytes in the PBMCs of HLA-A2^+^ donor. The proportion of IFN-*γ*-secreting CD8^+^ T cells are indicated as percentage in the bidimenional flow cytometry diagram. (**A**) Effector cells with HepG2 cells; (**B**) effector cells only.

To estimate the frequency of HCA661-specific CTLs after stimulation with transfected DCs, intracellular flow cytometry (CytoSpot) assay and ELISPOT assay were used. In the CytoSpot assay, 6.4% of PBMCs stimulated with Ad661-transfected DCs stained positive with both anti-IFN-*γ* and anti-CD8 mAbs after being cocultured with irradiated HepG2 cells for 6 h in the presence of monensin (Figure 4A

). In contrast, the PBMCs stimulated with QBI-Infect+-transfected DCs or DCs alone showed only 4.1 and 3.2% positive staining with both anti-IFN-*γ* and anti-CD8 mAbs, respectively (Figure 4A). The percentage of HCA661-specifc CTLs was calculated as the number of IFN-*γ*^+^CD8^+^ effector T cells reactivated with HepG2 cells (Figure 4A) deducting the number of IFN-*γ*^+^ CD8^+^T cells in the cultures without reactivation with HepG2 cells (Figure 4B). The results showed that PBMCs presensitised with Ad661-transduced DCs exhibited 2.3% HCA661-specific CTLs, which is approximately five times more than that of PBMCs presensitised with QBI-Infect+-transduced DCs or DCs alone (0.1 and 0.4% HCA661-specific CTLs, respectively).

**Figure 5:**
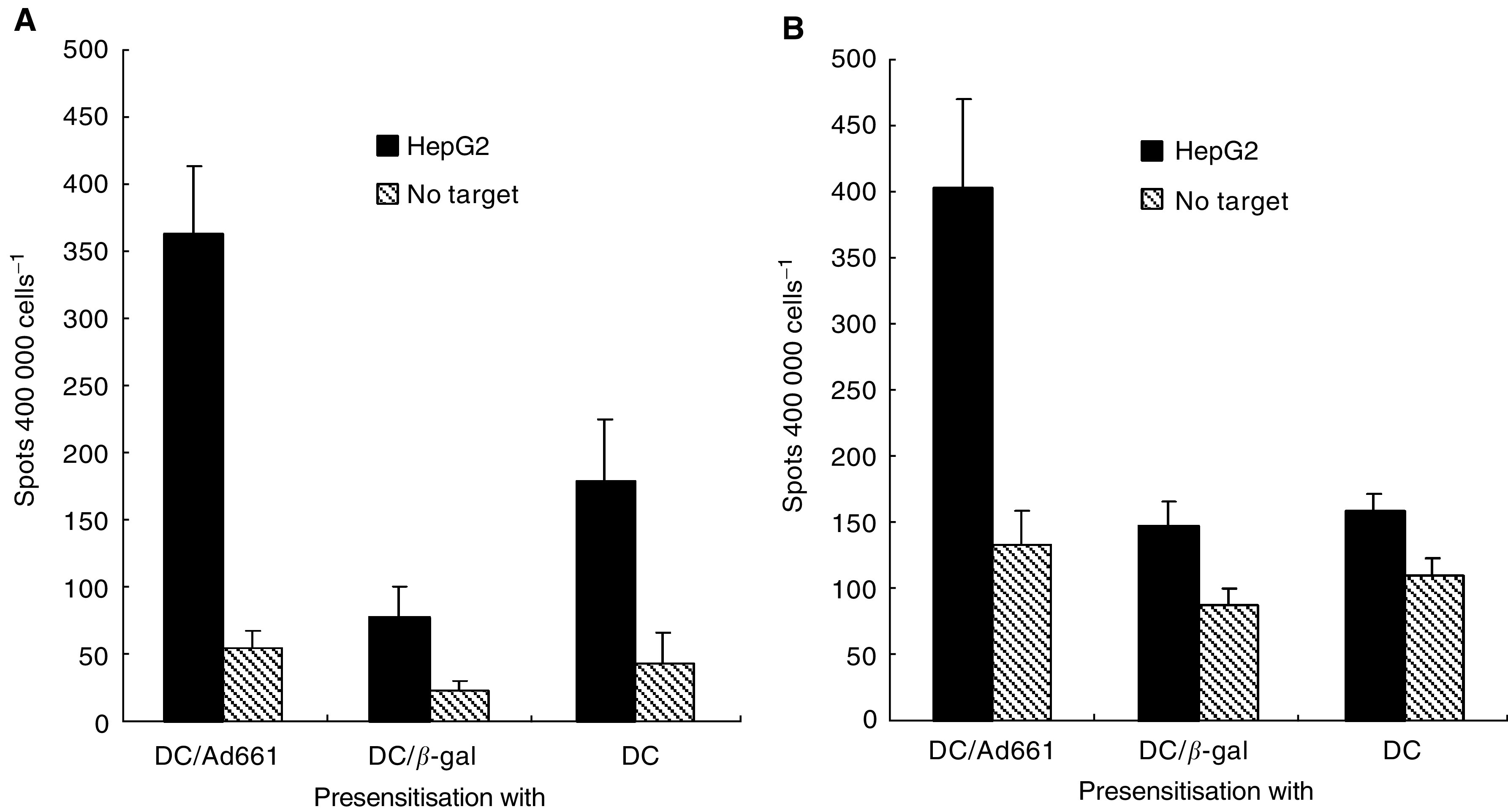
Presensitisation of PBMCs from HLA-A2^+^ donors (**A** and **B**). Peripheral blood mononuclear cells were presensitised with irradiated DCs transduced by Ad661, QBI-Infect+ (*β*-gal) or DCs alone and tested against the HLA-A2^+^ target cell, HepG2 on day 19 using ELISPOT. Results represent the mean number of spots per 40 000 effector PBMCs in duplicate wells, with error bars indicating standard deviation. Differences between paired cultures were compared by two-tailed Student's *t-*test for paired samples (*P*<0.05).

The HCA661-specific T cells have also been identified reliably with an IFN-*γ* ELISPOT assay in PBMCs from HLA-A2 healthy positive donors primed with adenovirus-transfected DCs. The results show that the PBMCs primed with Ad661-transfected DCs have approximately 400 IFN-*γ* spots, which is four times more spots than on PBMCs primed with QBI-Infect+ transfected DCs or DCs alone (Figure 5A and B

). These data indicate that Ad661 transfected DCs can stimulate PBMCs to generate IFN-*γ*-secreting cells, which according to the results of intracellular flow cytometry are CD8+ T cells (Figure 4). These findings demonstrate the presence of HCA661 specific CD8+ T cells in PBMCs after activativion by Ad661-transfected DCs.

## DISCUSSION

In the last few years, identification of tumour-associated antigens (TAAs) (Jäger *et al*, 2001) has prompted the development of different strategies for antitumour vaccination, aimed at inducing specific recognition of TAA-derived peptides in order to elicit a persistent immune memory that may eliminate residual tumour cells and protect recipients from relapses (Bocchia *et al*, 2000). Researchers have identified tumour antigens that are expressed only on cancer cells, and not on normal cells except for germ cells. These TAAs have been used as targets in immunotherapy. However, there are some problems associated with TAA peptide-based immunotherapy. TAA peptides are presented by certain types of HLA molecules (Sadanaga *et al*, 2001), Therefore, HLA typing must be carried out before using a particular TAA peptide-based immunotherapy.

The present study explores the use of DCs transfected with a recombinant adenovirus for the treatment of HCC. This approach can overcome the problem of HLA restriction in TAA-based immunotherapy. Using viral vectors with genes encoding the full-length tumour antigens to transfect autologous DCs to activate both CD4+ and CD8+ T cells makes the typing of HLA haplotypes unnecessary. In principle, this approach could lead to an anti-HCC vaccine. Dendritic cells could be generated from blood of patients and then transfected by recombinant adenovirus Ad661. The transfected DCs could then activate patients' T cells to target the cancer cells in the patient, without concern for HLA matching.

It has been observed that HCC cells express multiple types of tumour antigens at the mRNA level, including members of MAGE family and NY-ESO-1 (Yamashita *et al*, 1996; Tahara *et al*, 1999). The protein of HCA661 is immunogenic and capable of inducing an antibody response, as it was screened by SEREX from the serum of an HCC patient (Wang *et al*, 2002). HCA661 is a CT antigen, as it does not normally express in human tissues (except testis) and it expresses at 30% in HCC samples (Wang *et al*, 2002). The potential of SEREX-defined CT antigens to induce cellular immunity despite a low frequency of antibodies in allogenic sera has been confirmed (Wang *et al*, 2002). The SEREX-defined NY-ESO-1 can induce a CTL response and the CTL-defined MAGEA-1 has also been identified by SEREX (Chen *et al*, 1998; Jager *et al*, 2000). This suggests that SEREX-cloned antigens may contain T- and B-cell epitopes. These epitopes are capable of inducing either Ab or CTL responses. In this study, the capacity of HCA661 to elicit a CTL response has been determined using a recombinant adenovirus to transduce the full-length HCA661 into DCs.

One reason why the immune system cannot eradicate cancer is that cancer cells lack the ability to stimulate T-cell activation. Mature and activated DCs are potent antigen-presenting cells (APCs) (Chan *et al*, 2002), which express MHC class I, class II, costimulatory and adhesion molecules that provide the two necessary signals for the activation of T-cell populations (Romani *et al*, 1989). Recombinant adenovirus technology has been widely employed in gene therapy and for delivering genes into primary cell types including murine DCs (Ribas *et al*, 1997; Song *et al*, 1997; Wan *et al*, 1997). In humans, adenoviral vectors expressing immunostimulatory cytokines or tumour antigens have recently been used to elicit antitumour immunity in gene therapy (Molnar-Kimber *et al*, 1998; Schuler *et al*, 1998). However, mature DCs are less susceptible to transfection than immature DCs (Zhong *et al*, 1999), due to reduced expression of *α*_v_*β*_5_ integrin (Albert *et al*, 1998) that mediates the uptake of adenoviruses (Wickham *et al*, 1993). In our study, we transfected immature DCs with recombinant adenovirus and then induced the DCs to mature by using the maturation agent. We found that the mRNA of HCA661 was expressed in the Ad661-transfected DCs (Figure 3D), and ELISPOT and CytoSpot assays (Figures 4 and 5) demonstrated that the mature DCs could stimulate HLA-A2^+^ CD8+ T cells to target the HepG2 cells, which are both HLA-A2 and HCA661 positive (Figure 3C). Our transfected DCs expressed the HCC tumour-associated antigen HCA661 as well as deleted the fragments of adenovirus capable of inducing neutralising antibodies against the adenovirus. These Ad661-transduced DCs have potential application for use in immunotherapy.

The development of neutralising antibodies against adenoviral antibodies following injection of viral vectors is a major problem in recombinant adenoviral immunotherapy (Christ *et al*, 1997). This problem can be avoided by *in vitro* transduction of DCs. Since the adenovirus would be internalised, the chance of direct exposure of viral envelope to the host would be reduced, and therefore would not be available to B cells.

We found that recombinant adenovirus-mediated transduction does not inhibit DC maturation (Figure 2). Compared with the DCs matured by maturation agents only, the adenovirus-transfected DCs showed a slightly higher expression of DC maturation marker CD83. The adenovirus may enhance the maturation effect of MCM and poly [I] : poly [C]. Poly [I] : poly [C] are synthetic viral polyribonucleotide analogues that mimic a viral danger signal and are recognised by TOLL 3 receptors on DCs (Verdijk *et al*, 1999). Thus, the adenovirus may enhance the viral danger signal to DCs for maturation. However, the adenovirus itself does not drive the maturation of immature DCs without MCM and poly [I] : poly [C] (data not shown). In human monocyte-derived DCs, mature DCs are less susceptible to transfection than immature DCs, because immature DCs selectively express the *α*_v_*β*_5_ integrin that mediates the uptake of adenoviruses (Zhong *et al*, 1999). Our findings suggest that *in vitro* transfection of immature DCs with adenovirus does not affect its further development into mature DCs. Our adenovirus-transfected DCs showed a greater than 90% transfection efficiency for the reporter gene expression (Figure 1B) and also displayed mature DCs phenotypes (Figures 2A and 3B).

We conclude that human monocyte-derived immature DCs transfected with recombinant adenovirus Ad661 *ex vivo* and developed into mature DCs with MCM and poly [I] : poly [C] were found to express HCA661 and to induce HCA661-specific CTL reaction. Ad661 may be a promising compound for use in the immunotherapy of HCC.
